# Ultrasound-guided microwave ablation versus conventional surgery for Granulomatous Lobular Mastitis: A two-center retrospective comparative stud

**DOI:** 10.1371/journal.pone.0351776

**Published:** 2026-06-26

**Authors:** Yinfeng Lin, Zhipeng Hong, Wangmei Xie, Aijun Lu, Xiuping Wu, Debo Chen

**Affiliations:** 1 The School of Clinical Medicine, Fujian Medical University, Fuzhou, P.R.China; 2 Department of Breast Surgery, Zhangzhou Zhengxing Hospital, Zhangzhou, P.R.China; 3 Department of Breast Surgery, Affiliated Quanzhou First Hospital of Fujian Medical University, Quanzhou, P.R.China; 4 Department of Gastroenterology, Hui’an Maternal and Child Health Hospital, Quanzhou, P.R.China; Universidad Santiago de Cali, COLOMBIA

## Abstract

**Background:**

Granulomatous lobular mastitis (GLM) is a chronic inflammatory breast disease that causes substantial pain, cosmetic deformity, and psychosocial distress. Evidence directly comparing ultrasound-guided microwave ablation (MWA) with conventional surgery (CS) remains limited.

**Methods:**

We conducted a two-center retrospective cohort study of consecutive women with core needle biopsy-confirmed GLM treated between March 2020 and February 2025. Treatment allocation was non-random and reflected routine clinical decision-making. The primary outcomes were overall cure and recurrence. Secondary outcomes included perioperative resource utilization and patient-reported outcomes assessed with a BREAST-Q domain-based questionnaire adapted for GLM follow-up. Baseline balance was described with both P values and standardized mean differences (SMDs).

**Results:**

Among 233 patients (MWA, n = 102; CS, n = 131), overall cure was achieved in 97.06% and 96.18% of patients, respectively (risk difference, 0.88 percentage points; 95% CI, −3.76 to 5.51; P = 1.000). Recurrence occurred in 4.90% of the MWA group and 3.82% of the CS group (risk difference, 1.09 percentage points; 95% CI, −4.24 to 6.41; P = 0.751) during a median follow-up of 18.5 months. Compared with CS, MWA was associated with lower estimated blood loss, shorter procedure time, lower postoperative drainage burden, and shorter hospitalization (all *P* < 0.001), while total hospitalization cost did not differ significantly. Questionnaire scores favored MWA for sexual well-being, breast satisfaction, and scar satisfaction, whereas psychological well-being, chest physical well-being, and satisfaction with the medical team were similar between groups. SMDs indicated some baseline imbalance, particularly for multiple-quadrant involvement and ultrasound lesion size.

**Conclusions:**

In this retrospective observational comparison, MWA and CS were associated with similarly high cure rates and low recurrence. MWA was additionally associated with lower perioperative burden and better breast- and scar-related satisfaction. Prospective studies with standardized co-interventions, confounding adjustment, and validated patient-reported outcome assessment are warranted.

## Introduction

Granulomatous lobular mastitis (GLM), also termed idiopathic granulomatous mastitis in many clinical series, is an uncommon chronic inflammatory disease of the breast that predominantly affects women of reproductive age [1]. Histopathologically, it is characterized by noncaseating granulomatous inflammation centered on the breast lobules. The disease is often difficult to diagnose and manage because its clinical presentation overlaps with abscess, plasma cell mastitis, tuberculous mastitis, and, less commonly, breast malignancy [1–3].

Treatment remains controversial. Reported strategies include observation, antibiotics, corticosteroids or other immunomodulatory agents, drainage procedures, wide local excision, and, more recently, minimally invasive thermal ablation. Conventional surgery (CS) offers tissue removal and immediate local control in selected cases, but may require relatively large incisions, scar formation, contour deformity, and prolonged wound care when disease is extensive or multifocal [2–5].

Ultrasound-guided microwave ablation (MWA) has emerged as a minimally invasive alternative that can target inflammatory lesions through a percutaneous approach while limiting tissue excision. Early case series and comparative studies suggest encouraging disease control together with favorable cosmetic outcomes [6–9]. However, the available evidence remains largely retrospective, and the clinical value of MWA relative to conventional surgery, particularly in terms of perioperative burden and patient-reported outcomes, has not been fully characterized.

## Materials and methods

### Study design and setting

We conducted a retrospective comparative study of patients with GLM treated between March 2020 and February 2025 at the Affiliated First Hospital of Quanzhou, Fujian Medical University and the Breast Center of Zhangzhou Zhengxing Hospital. The study was approved by the ethics committees of both hospitals (ethics approval number: Quan-Yi-Lun[2025]K089). This retrospective study was conducted in full accordance with the National Guidelines for Ethical Review of Biomedical Research Involving Human Subjects (China, 2016) and the Declaration of Helsinki (2013 revision). Written informed consent was obtained from all enrolled patients or their authorized family members.

Because the study was retrospective, no prospective a priori sample size calculation was performed. Instead, all consecutive eligible patients treated during the predefined study period were included. The manuscript was revised in line with STROBE reporting principles for observational studies [10].

### Patient selection and diagnostic workflow

Inclusion criteria were: (1) female patients over 18 years of age; (2) GLM confirmed on pre-treatment by core needle biopsy; (3) available pre-treatment breast ultrasonography; (4) availability for follow-up; (5) complete clinical data. Exclusion criteria were: (1) male sex; (2) concomitant malignancy; (3) severe cardiac, hepatic, or pulmonary dysfunction; (4) incomplete clinical data; and (5) inability to complete follow-up.

The pathological diagnosis of GLM required noncaseating granulomatous lobulitis centered on breast lobules, typically composed of epithelioid histiocytes and multinucleated giant cells, with or without neutrophilic microabscesses, after exclusion of malignancy and other specific granulomatous diseases [1,2,11]. For patients in the CS group, postoperative excision pathology was additionally available. For patients in the MWA group, diagnosis relied on the pre-ablation core needle biopsy because MWA does not generate an excision specimen.

Alternative infectious causes, including tuberculous mastitis, nontuberculous mycobacterial infection, fungal infection, and other specific infectious mastitides, were excluded through clinicopathologic review and microbiologic testing when clinically indicated in routine care. However, the retrospective database did not uniformly preserve the exact microbiologic pathway for every patient, such as whether acid-fast stains, mycobacterial culture, polymerase chain reaction, or Xpert MTB/RIF were performed in each case. Likewise, systematic documentation of extramammary tuberculosis screening was not available for the analytic dataset. These issues are therefore acknowledged as limitations rather than reported as standardized study procedures.

### Treatment allocation

Treatment assignment was non-random. After histologic confirmation of GLM, the attending breast surgeon discussed available local treatment options with the patient. The final choice between MWA and CS reflected routine clinical decision-making, including lesion extent, multifocality, lesion location relative to the skin and nipple-areolar complex, the anticipated feasibility of complete percutaneous coverage, the expected cosmetic effect, anesthesia considerations, patient preference, and local center practice. Accordingly, this study should be interpreted as an observational comparison rather than a randomized efficacy trial.

### Clinical data collection

Demographic and clinical data were collected, including age, marital status, pregnancy history, body mass index (BMI), history of breast trauma, comorbidities, psychotropic medication use, symptom duration, fever, lesion location, skin redness, nipple discharge, nipple retraction, mass size, and laboratory parameters (prolactin, white blood cell count, neutrophil count, C-reactive protein, triglycerides, total cholesterol, and low-density lipoprotein). Ultrasound characteristics were documented, including lesion number, mass size, internal echo pattern, border morphology, blood flow signals, and ipsilateral axillary lymph node changes (maximum diameter, aspect ratio, cortical thickening, and blood flow). Data were accessed for research purposes in 01/04/2025.

### Procedures

MWA was performed under real-time ultrasound guidance using a microwave power setting of 25 W under local anesthesia. The ablation was performed systematically from the lesion margin toward the center and from deep to superficial portions until the targeted area showed complete hyperechoic coverage on ultrasound. The goal was to achieve local ablation of all clinically and sonographically relevant inflammatory tissue while preserving as much surrounding breast tissue as possible.

CS was performed under general anesthesia through a periareolar or radial incision chosen according to lesion location and surgical accessibility. Inflammatory tissue was excised according to routine surgical judgment, and glandular flap techniques were used in selected cases to reduce contour deformity. Drain placement was determined by the operating surgeon.

Patients in both groups could receive adjunctive conservative management before or after the procedure according to routine clinical care, such as wound care, analgesia, drainage-related care, and antimicrobial or anti-inflammatory treatment when clinically indicated. Because these co-interventions were not standardized and the revision materials did not preserve patient-level regimen details, they are acknowledged as a potential source of residual confounding rather than as protocolized identical treatment.

### Outcome measures

The primary clinical outcomes were overall cure and recurrence. Complete radiologic remission was defined as complete clinical and imaging resolution without evidence of active residual disease during follow-up. Clinical cure was defined as resolution of symptoms and inflammatory signs, with no palpable lesion, despite minor non-progressive residual findings on imaging. Overall cure was calculated as the proportion of patients who achieved either complete radiologic remission or clinical cure. Uncured disease referred to persistent symptoms, signs, or active imaging abnormalities requiring ongoing treatment.

Recurrence was defined as reappearance of compatible GLM manifestations at a previously controlled site after an initial response during follow-up, supported by imaging findings and, when re-intervention was undertaken, pathology. Recurrence rate was calculated as the number of recurrent cases divided by the total number of treated patients.

Perioperative outcomes included estimated blood loss, procedure duration, postoperative drainage burden, length of hospitalization, and total hospitalization cost in Renminbi (RMB). Procedure time was defined as the interval from skin puncture or surgical incision to completion of wound dressing. Estimated blood loss was abstracted from the procedure or anesthesia record. Length of stay was defined as the interval from admission to discharge.

### Patient-reported outcome assessment

Patient-reported outcomes were evaluated at follow-up using a questionnaire derived from licensed BREAST-Q domains relevant to psychosocial well-being, sexual well-being, breast satisfaction, scar satisfaction, chest physical well-being, and satisfaction with the medical team. In the original retrospective database, these data were stored as domain-level Likert summaries rather than as fully converted QScore outputs. Accordingly, results are reported here as mean item scores on the original 1–5 scale rather than as standard Rasch-transformed 0–100 BREAST-Q scores (Use of this Questionnaire, authored by Drs. Klassen, Pusic and Cano, was made under license from Memorial Sloan Kettering Cancer Center, New York, USA). To avoid overstating validation, the revised manuscript refers to this instrument as a BREAST-Q domain-based questionnaire adapted for GLM follow-up.

The questionnaire was administered during postoperative follow-up, and domain analyses were based on available recorded responses. Because item-level response files were not preserved in the revision materials, missingness by item or domain could not be fully reconstructed and no additional imputation was performed. The reported domain scores therefore reflect the available retrospective records only.

### Statistical analysis

Statistical analysis was performed using IBM SPSS Statistics 27.0.1 (IBM Corp., Armonk, NY, USA). Categorical variables were presented as frequencies and percentages, and continuous variables as means with standard deviations or medians with interquartile ranges as appropriate. Comparisons between groups were performed using the chi-square test or Fisher’s exact test for categorical variables and the Student’s t-test or Mann-Whitney U test for continuous variables, as appropriate. All tests were two-sided, and *P* < 0.05 was considered statistically significant.

To improve interpretability in this non-randomized comparison, effect estimates with 95% confidence intervals (CIs) were calculated for the primary binary outcomes. Baseline balance was additionally described using standardized mean differences (SMDs), with larger absolute values interpreted as greater evidence of meaningful imbalance [12,13]. Because the present report is observational and unadjusted, the results are presented as associations rather than causal treatment effects.

## Results

### Study cohort and baseline balance

A total of 233 patients met the eligibility criteria, including 102 treated with MWA and 131 treated with CS. Baseline demographic, clinical, laboratory, and ultrasound findings are summarized in Tables 1–3. Conventional hypothesis testing did not identify statistically significant between-group differences for most baseline variables. However, SMDs suggested some clinically relevant imbalance, which is consistent with the non-random treatment allocation described above.

**Table 1:** Baseline characteristics of patients with granulomatous lobular mastitis treated with microwave ablation (MWA) versus conventional surgery (CS).

Parameter	Total	MWA	CS	P value
*Age* (*years*), *median*	33	34	32	0.156
*Age* (*years*), *mean*	33.31	34.17	32.63	
*Marital status*: *single*	4 (1.72%)	1 (0.98%)	3 (2.29%)	0.633
*Marital status*: *married*	229 (98.28%)	101 (99.02%)	128 (97.71%)	
*Pregnancy history*: *no*	7 (3.00%)	1 (0.98%)	6 (4.58%)	0.140
*Pregnancy history*: *yes*	226 (97.00%)	101 (99.02%)	125 (95.42%)	
*Number of pregnancies among women with pregnancy history*: 1	98 (43.36%)	44 (43.56%)	54 (43.20%)	
*Number of pregnancies among women with pregnancy history*: 2	119 (52.66%)	53 (52.48%)	66 (52.80%)	
*Number of pregnancies among women with pregnancy history*: ≥ 3	9 (3.98%)	4 (3.96%)	5 (4.00%)	
*BMI* < 18.5 *kg*/*m*²	8 (3.43%)	4 (3.92%)	4 (3.05%)	0.399
*BMI* 18.5 *to* <24.0 *kg*/*m*²	101 (43.35%)	49 (48.04%)	52 (39.69%)	
*BMI* 24.0 *to* <28.0 *kg*/*m*²	69 (29.61%)	30 (29.41%)	39 (29.77%)	
*BMI* ≥ 28.0 *kg*/*m*²	55 (23.61%)	19 (18.63%)	36 (27.48%)	
*History of breast trauma*: *no*	206 (88.41%)	91 (89.22%)	115 (87.79%)	0.838
*History of breast trauma*: *yes*	27 (11.59%)	11 (10.78%)	16 (12.21%)	
*Comorbidity*: *no*	225 (96.57%)	99 (97.06%)	126 (96.18%)	0.356
*Comorbidity*: *yes*	8 (3.43%)	3 (2.94%)	5 (3.82%)	
*Psychotropic medication*: *no*	226 (97.00%)	101 (99.02%)	125 (95.42%)	0.140
*Psychotropic medication*: *yes*	7 (3.00%)	1 (0.98%)	6 (4.58%)	
*Symptom duration* <3 *months*	180 (77.25%)	79 (77.45%)	101 (77.10%)	0.815
Symptomduration3−−6months	33 (14.16%)	15 (14.71%)	18 (13.74%)	
*Symptom duration* >6 *months*	20 (8.58%)	8 (7.84%)	12 (9.16%)	

**Table 2:** Clinical characteristics and laboratory findings in patients with granulomatous lobular mastitis treated with MWA versus CS.

Parameter	Total	MWA	CS	P value
*Fever*: *no*	221 (94.85%)	98 (96.08%)	123 (93.89%)	0.558
*Fever*: *yes*	12 (5.15%)	4 (3.92%)	8 (6.11%)	
*Mass quadrant location*: *upper outer*	40 (17.17%)	23 (22.55%)	17 (12.98%)	0.066
*Mass quadrant location*: *lower outer*	16 (6.87%)	9 (8.82%)	7 (5.34%)	
*Mass quadrant location*: *upper inner*	36 (15.45%)	17 (16.67%)	19 (14.50%)	
*Mass quadrant location*: *lower inner*	23 (9.87%)	12 (11.76%)	11 (8.40%)	
*Mass quadrant location*: *multiple quadrants*	118 (50.64%)	41 (40.20%)	77 (58.78%)	
*Skin redness*: *no*	103 (44.21%)	39 (38.24%)	64 (48.85%)	0.113
*Skin redness*: *yes*	130 (55.79%)	63 (61.76%)	67 (51.15%)	
*Nipple discharge*: *no*	104 (44.64%)	48 (47.06%)	56 (42.75%)	0.595
*Nipple discharge*: *yes*	129 (55.36%)	54 (52.94%)	75 (57.25%)	
*Nipple retraction*: *no*	159 (68.24%)	72 (70.59%)	87 (66.41%)	0.571
*Nipple retraction*: *yes*	74 (31.76%)	30 (29.41%)	44 (33.59%)	
*Mass size* (*cm*), *median*	5	5	6	0.140
*Mass size* (*cm*), *mean*	5.743	5.442	5.978	
*Elevated prolactin*: *no*	201 (86.27%)	93 (91.18%)	108 (82.44%)	0.058
*Elevated prolactin*: *yes*	32 (13.73%)	9 (8.82%)	23 (17.56%)	
*Elevated WBC count*: *no*	119 (51.07%)	47 (46.08%)	72 (54.96%)	0.189
*Elevated WBC count*: *yes*	114 (48.93%)	55 (53.92%)	59 (45.04%)	
*Elevated neutrophil ratio*: *no*	164 (70.39%)	70 (68.63%)	94 (71.76%)	0.665
*Elevated neutrophil ratio*: *yes*	69 (29.61%)	32 (31.37%)	37 (28.24%)	
*Elevated CRP*: *no*	172 (73.82%)	81 (79.41%)	91 (69.47%)	0.099
*Elevated CRP*: *yes*	61 (26.18%)	21 (20.59%)	40 (30.53%)	
*Elevated triglycerides*: *no*	173 (74.25%)	78 (76.47%)	95 (72.52%)	0.547
*Elevated triglycerides*: *yes*	60 (25.75%)	24 (23.53%)	36 (27.48%)	
*Elevated total cholesterol*: *no*	220 (94.42%)	95 (93.14%)	125 (95.42%)	0.568
*Elevated total cholesterol*: *yes*	13 (5.58%)	7 (6.86%)	6 (4.58%)	
*Elevated LDL*: *no*	221 (94.85%)	99 (97.06%)	122 (93.13%)	0.237
*Elevated LDL*: *yes*	12 (5.15%)	3 (2.94%)	9 (6.87%)	

**Table 3:** Ultrasonographic findings in patients with granulomatous lobular mastitis treated with MWA versus CS.

Parameter	Total	MWA	CS	P value
*Number of lesions*: 1	116 (49.79%)	50 (49.09%)	66 (50.38%)	0.882
*Number of lesions*: 2	58 (24.89%)	27 (26.47%)	31 (23.66%)	
Numberoflesions:≥3	59 (25.32%)	25 (24.51%)	34 (25.95%)	
*Ultrasound lesion size* <2 *cm*	8 (3.43%)	2 (1.96%)	6 (4.58%)	0.080
Ultrasoundlesionsize2−-5cm	130 (55.79%)	65 (63.73%)	65 (49.62%)	
*Ultrasound lesion size* >5 *cm*	95 (40.77%)	35 (34.31%)	60 (45.80%)	
*Internal echo*: *low*	124 (53.22%)	58 (56.86%)	66 (50.38%)	0.574
*Internal echo*: *anechoic*	14 (6.01%)	5 (4.90%)	9 (6.87%)	
*Internal echo*: *mixed*	95 (40.77%)	39 (38.24%)	56 (42.75%)	
*Border morphology*: *irregular*	160 (68.67%)	69 (67.65%)	91 (69.47%)	0.778
*Border morphology*: *regular*	73 (31.33%)	33 (32.35%)	40 (30.53%)	
*Internal blood flow signal*: *no*	60 (25.75%)	32 (31.37%)	28 (21.37%)	0.097
*Internal blood flow signal*: *yes*	173 (74.25%)	70 (68.63%)	103 (78.63%)	
*Ipsilateral axillary lymphadenopathy*: *no*	69 (29.61%)	33 (32.35%)	36 (27.48%)	0.470
*Ipsilateral axillary lymphadenopathy*: *yes*	164 (70.39%)	69 (67.65%)	95 (72.52%)	
Maximumlymphnodediameter<1cm*	9 (5.49%)	4 (5.80%)	5 (5.26%)	0.350
Maximumlymphnodediameter1−-2cm*	112 (68.29%)	43 (62.32%)	69 (72.63%)	
Maximumlymphnodediameter>2cm*	43 (26.22%)	22 (31.88%)	21 (22.11%)	
Lymphnodeaspectratio<0.5*	98 (59.76%)	38 (55.07%)	60 (63.16%)	0.335
Lymphnodeaspectratio0.5−−1.0*	66 (40.24%)	31 (44.93%)	35 (36.84%)	
Corticalthickening:no*	119 (72.56%)	52 (75.36%)	67 (70.53%)	0.595
Corticalthickening:yes*	45 (27.44%)	17 (24.64%)	28 (29.47%)	
Lymphnodebloodflowsignal:no*	14 (8.54%)	9 (13.04%)	5 (5.26%)	0.094
Lymphnodebloodflowsignal:yes*	150 (91.46%)	60 (86.96%)	90 (94.75%)	

* Percentages for lymph-node-related variables are calculated among patients with ipsilateral axillary lymphadenopathy (n = 164), not the full cohort.

In particular, the CS group showed a higher proportion of multiple-quadrant involvement and a greater proportion of lesions larger than 5 cm on ultrasound, whereas the MWA group had somewhat higher frequencies of skin redness and lower frequencies of elevated prolactin and CRP. Selected SMDs are presented in S1 Table. These findings support cautious interpretation of any apparent between-group differences as observational rather than causal.

### Perioperative outcomes

**Fig 1:**
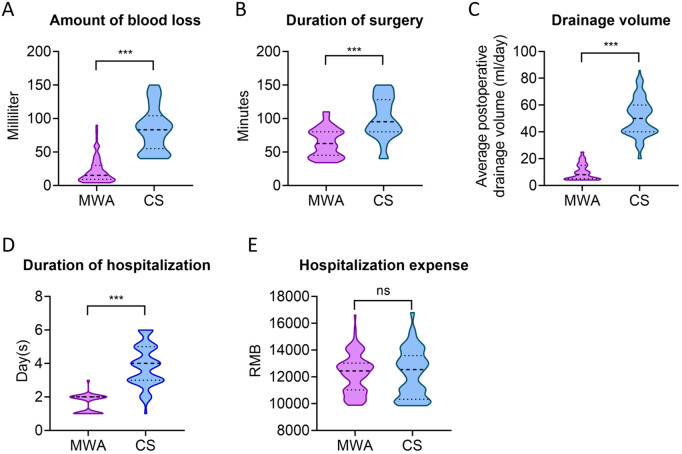
Perioperative outcomes comparing MWA versus CS in GLM treatment. **(A)** Estimated blood loss. **(B)** Procedure duration. **(C)** Average postoperative drainage burden. **(D)** Length of hospitalization. **(E)** Total hospitalization expenses (RMB). Lower blood loss, procedure time, drainage burden, and hospitalization duration favored MWA, whereas total cost did not differ significantly. ***P<0.001, ns, not significant.

Compared with CS, MWA was associated with significantly lower estimated blood loss, shorter procedure duration, lower postoperative drainage burden, and shorter hospitalization (all *P* < 0.001; Fig 1A–D). Total hospitalization cost did not differ significantly between groups (Fig 1E). Taken together, these findings indicate that MWA was associated with a lower perioperative resource burden in routine practice.

### Primary clinical outcomes

Median follow-up for the overall cohort was 18.5 months. Overall cure was achieved in 99 of 102 patients (97.06%) in the MWA group and 126 of 131 patients (96.18%) in the CS group. The absolute risk difference was 0.88 percentage points (95% CI, −3.76 to 5.51), and the corresponding risk ratio was 1.01 (95% CI, 0.96 to 1.06; Fisher exact P = 1.000).

**Fig 2:**
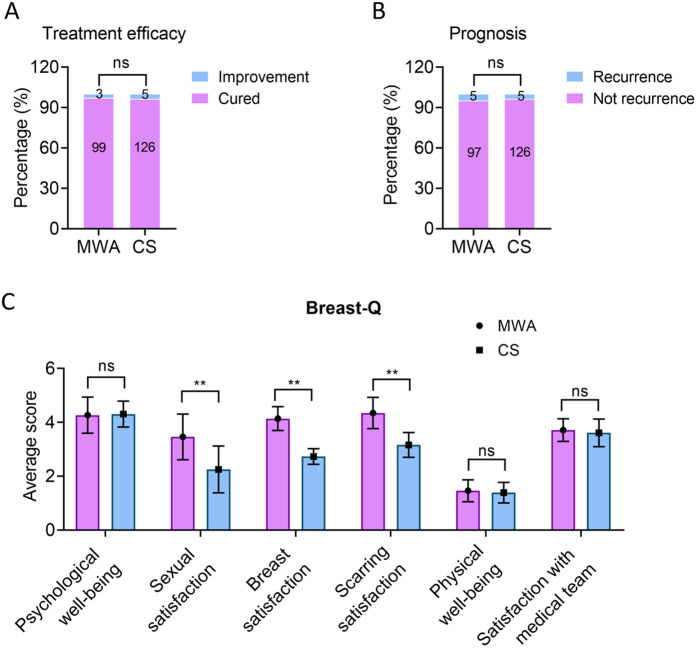
Comparison of primary clinical outcomes and patient-reported outcomes between MWA and CS. **(A)** Treatment efficacy showing percentage of patients cured versus improved in MWA and CS groups, with no significant difference between treatments (ns, not significant). **(B)** Prognosis showing recurrence rates between MWA and CS groups, with comparable outcomes (ns, not significant). **(C)** BREAST-Q scores comparing patient satisfaction across six domains between MWA and CS groups. While psychological well-being, physical well-being, and satisfaction with medical team showed no significant differences (ns, not significant), MWA demonstrated significantly higher scores for sexual satisfaction, breast satisfaction, and scarring satisfaction (**P<0.01). Error bars represent standard deviation.

Recurrence during follow-up occurred in 5 of 102 patients (4.90%) after MWA and 5 of 131 patients (3.82%) after CS. The absolute risk difference was 1.09 percentage points (95% CI, −4.24 to 6.41), and the corresponding risk ratio was 1.28 (95% CI, 0.38 to 4.32; Fisher exact P = 0.751). These estimates indicate that no statistically significant between-group difference was observed for either overall cure or recurrence in this cohort (Table 4; Fig 2A–B).

**Table 4:** Primary clinical outcome effect estimates comparing MWA with CS.

Outcome	MWA	CS	Risk difference (95% CI)	Risk ratio (95% CI)	P value
*Overall cure*	99/102 (97.06%)	126/131 (96.18%)	0.88% (−3.76 to 5.51)	1.01 (0.96–1.06)	1.000
*Recurrence*	5/102 (4.90%)	5/131 (3.82%)	1.09% (−4.24 to 6.41)	1.28 (0.38–4.32)	0.751

Risk differences are expressed in percentage points. P values were calculated with Fisher’ exact test.

### Patient-reported outcomes

The BREAST-Q domain-based questionnaire showed no clear between-group difference in psychological well-being, chest physical well-being, or satisfaction with the medical team. In contrast, patients treated with MWA reported higher sexual well-being, greater breast satisfaction, and better scar satisfaction than patients treated with CS (Fig 2C). Given the non-standard 1–5 scoring framework used in the retrospective database, these findings should be interpreted as comparative patient-reported patterns within this study rather than as directly comparable formal BREAST-Q QScores.

### Representative case

**Fig 3:**
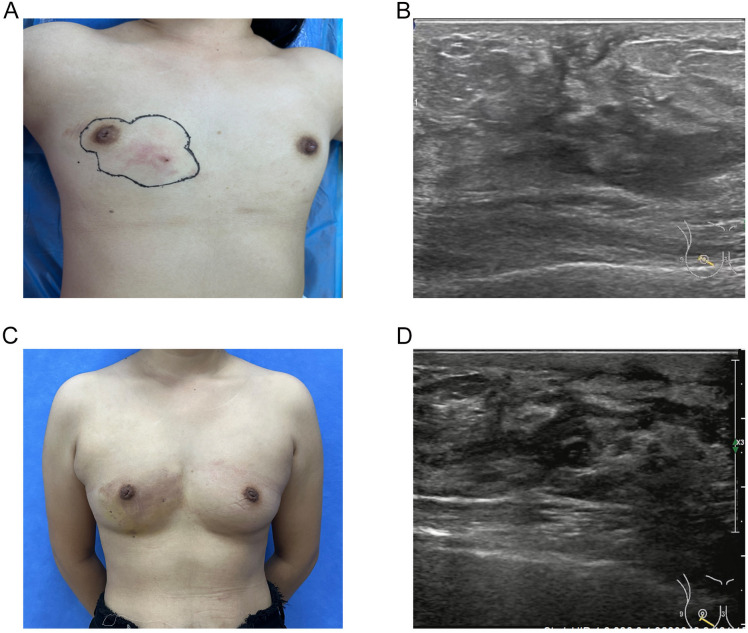
Microwave ablation for GLM in a 31-year-old woman. **(A)** Preoperative appearance of the right breast with outlined lesion area. **(B)** Preoperative ultrasound showing a hypoechoic lesion classified as BI-RADS 3. **(C)** Postoperative appearance at follow-up demonstrating excellent cosmetic outcome with preserved breast contour and no visible scarring. **(D)** Postoperative ultrasound showing the treated area with resolved inflammatory changes. This case illustrates the minimal invasiveness and superior aesthetic outcomes achievable with ultrasound-guided MWA for GLM. Reprinted from original research under a CC BY license, with permission from Debo Chen, original copyright 2026.

Case 1. A 31-year-old woman presented with a right breast mass discovered one month prior to evaluation (Fig 3A). Ultrasound examination revealed a hypoechoic area classified as BI-RADS 3 (Fig 3B). Core needle biopsy confirmed GLM. The patient underwent ultrasound-guided MWA of the lesion. Fig 3C demonstrates postoperative outcome at follow-up showing excellent cosmetic results with preserved breast contour and no visible scarring. Corresponding ultrasound images confirm the diagnosis and treatment efficacy (Fig 3D).

**Fig 4:**
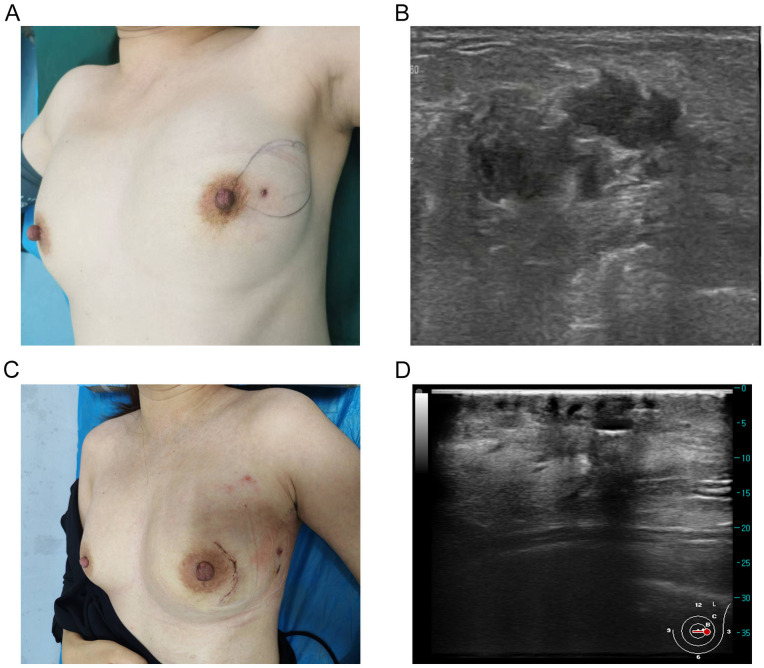
Conventional surgical treatment for GLM in a 35-year-old woman. **(A)** Preoperative appearance of the left breast with outlined lesion area. **(B)** Preoperative ultrasound showing a hypoechoic area with liquefaction, characteristic of granulomatous mastitis. **(C)** Postoperative appearance showing mild scarring but generally good cosmetic outcome following conventional surgical excision. **(D)** Postoperative ultrasound confirming effective resolution of the inflammatory lesion. This case demonstrates the standard surgical approach for GLM treatment with visible scarring as a typical consequence of the procedure. Reprinted from original research under a CC BY license, with permission from Debo Chen, original copyright 2026.

Case 2. A 35-year-old woman presented with a left breast mass and recurrent pain persisting for one year (Fig 4A). Ultrasound examination revealed a hypoechoic area with liquefaction, suggestive of granulomatous mastitis (Fig 4B). Core needle biopsy confirmed GLM. The patient underwent surgical excision and exploration of the lesion. Postoperative histopathology confirmed GLM with purulent inflammation. Fig 4C illustrates postoperative appearance with mild scarring but good overall cosmetic outcome. Corresponding ultrasound images confirm the effectiveness of conventional surgical management for this condition (Fig 4D).

## Discussion

This two-center retrospective comparison showed that both MWA and CS were associated with high overall cure rates and low recurrence during follow-up in patients with GLM. The absolute differences in cure and recurrence were small and the corresponding 95% CIs crossed the null, indicating that no statistically significant between-group difference was observed in the present cohort. At the same time, MWA was associated with less perioperative burden and more favorable patient-reported breast- and scar-related outcomes.

The domain-specific analysis of BREAST-Q scores provides critical insights into the patient experience across multiple dimensions. While psychological well-being remained equivalent between groups, the significantly higher sexual satisfaction and breast satisfaction scores in the MWA group represent meaningful quality-of-life advantages. These findings suggest that preservation of breast aesthetics and integrity with MWA positively impacts intimate relationships and body image—domains particularly important to the predominantly reproductive-age population affected by GLM. This advantage is further supported by Li et al. [8], who similarly reported enhanced sexual and psychosocial well-being following MWA compared to CS.

Perhaps most notably, the MWA group demonstrated significantly higher scarring satisfaction scores, representing a substantial aesthetic advantage in treatment selection. This finding aligns with Wang et al. [7], who reported superior cosmetic outcomes with minimal scarring for minimally invasive GLM approaches. The aesthetic benefit stems from MWA’s percutaneous approach requiring only a small incision for probe insertion, compared to longer incisions necessary for CS. Additionally, MWA preserves breast structural integrity by targeting only pathological tissue, whereas CS often requires more extensive tissue removal, potentially leading to contour irregularities and more prominent scarring [6,14]. Zhou et al. [6] established that smaller scar formation correlates with less psychological distress and better social functioning—explaining the connection between improved aesthetic outcomes and higher satisfaction scores.

Indeed, the recurrence rates observed in our cohorts (4.90% for MWA and 3.82% for CS) were statistically equivalent and notably lower than those reported in previous studies of GLM, which have documented recurrence rates ranging from 8% to 50% [5,15]. This comparative equivalence suggests that MWA achieves thorough elimination of inflammatory tissue comparable to surgical excision, while preserving surrounding healthy breast tissue integrity.

Our findings are broadly consistent with prior reports suggesting that ultrasound-guided MWA can provide effective local control for GLM while preserving breast appearance [8,9]. The clinical value of that preservation is important because GLM commonly affects young women for whom scar burden, breast contour, sexual well-being, and social confidence are highly relevant treatment outcomes. In practical terms, the present data suggest that MWA may be an attractive option for appropriately selected patients when percutaneous coverage is technically feasible and breast cosmesis is a priority.

This study was retrospective and non-randomized, and treatment allocation reflected routine clinical judgment rather than protocolized assignment. The SMD analysis indicates that some baseline imbalance was present, especially for disease distribution and lesion size, which raises the possibility of treatment-selection bias. Because no propensity score model or other confounding-adjusted model is presented here, causal interpretation should be avoided. The results should instead be read as an observational comparison of two real-world treatment pathways.

Several additional limitations deserve emphasis. First, center-level practice patterns and adjunctive medical or conservative treatments were not standardized or captured in sufficient detail to evaluate their influence on outcome. Second, infectious exclusion pathways, including tuberculosis- or mycobacteria-specific testing and extramammary tuberculosis assessment, were not uniformly archived in the analytic dataset. Third, patient-reported outcomes were stored as a BREAST-Q domain-based 1–5 Likert summary rather than as standard 0–100 QScores, which limits comparability with formal BREAST-Q studies. Fourth, treatment-related complications were not retrospectively graded with a standardized severity system, so the safety comparison should not be interpreted as exhaustive. Finally, the study included only patients with sufficiently complete records and follow-up, so selection bias cannot be excluded.

Despite these constraints, the study has several strengths. It includes a relatively large two-center cohort, evaluates both clinical and patient-centered outcomes, and presents effect sizes with confidence intervals for the primary endpoints. The addition of SMDs improves transparency about baseline balance and helps align the interpretation of the findings with the realities of retrospective comparative research. Future investigations should prospectively standardize co-interventions, capture modality-specific adverse events, use validated and fully scored patient-reported outcome instruments, and apply multivariable or propensity-based adjustment to address confounding more rigorously.

## Conclusion

In this retrospective observational study, ultrasound-guided MWA and CS were associated with similarly high cure rates and low recurrence in patients with GLM. MWA was further associated with lower perioperative burden and more favorable breast- and scar-related satisfaction. These findings support MWA as a promising minimally invasive option for selected patients, while prospective controlled studies remain necessary before stronger comparative claims can be made.

## Supporting information

S1 Table Selected clinically relevant baseline imbalances summarized with absolute standardized mean differences (SMDs). Absolute SMD values greater than 0.10 suggest potentially meaningful imbalance in this non-randomized comparison.
(DOCX)
