# Correction: Ultrasound-guided microwave ablation versus conventional surgery for Granulomatous Lobular Mastitis: A two-center retrospective comparative stud

**DOI:** 10.1371/journal.pone.0361493

**Published:** 2026-10-23

**Authors:** Yinfeng Lin, Zhipeng Hong, Wangmei Xie, Aijun Lu, Xiuping Wu, Debo Chen

The word “study” is misspelled in the article title. The correct title is: Ultrasound-guided microwave ablation versus conventional surgery for Granulomatous Lobular Mastitis: A two-center retrospective comparative study. The correct citation is: Lin Y, Hong Z, Xie W, Lu A, Wu X, Chen D (2026) Ultrasound-guided microwave ablation versus conventional surgery for Granulomatous Lobular Mastitis: A two-center retrospective comparative study. PLoS One 21(6): e0351776. https://doi.org/10.1371/journal.pone.0351776
