# Supplementary material for: Ultrasound-guided microwave ablation versus conventional surgery for Granulomatous Lobular Mastitis: A two-center retrospective comparative stud
Source: PLoS One. 2026 Jun 26;21(6):e0351776. doi: 10.1371/journal.pone.0351776 (PMC13308823; doi:10.1371/journal.pone.0351776)
Supplement: S1 Table — Absolute SMD values greater than 0.10 suggest potentially meaningful imbalance in this non-randomized comparison. (DOCX) [file pone.0351776.s001.docx]

Table S1: Selected clinically relevant baseline imbalances summarized with absolute standardized mean differences (SMDs).

| **Parameter** | **Microwave Ablation** | **Conventional Surgery** | **Absolute SMD** |
| --- | --- | --- | --- |
| Any pregnancy history | 101/102 (99.02%) | 125/131 (95.42%) | 0.220 |
| BMI ≥24.0 kg/m² | 49/102 (48.04%) | 75/131 (57.25%) | 0.185 |
| Symptom duration >3 months | 23/102 (22.55%) | 30/131 (22.90%) | 0.008 |
| Multiple-quadrant involvement | 41/102 (40.20%) | 77/131 (58.78%) | 0.378 |
| Skin redness | 63/102 (61.76%) | 67/131 (51.15%) | 0.215 |
| Elevated prolactin | 9/102 (8.82%) | 23/131 (17.56%) | 0.260 |
| Elevated CRP | 21/102 (20.59%) | 40/131 (30.53%) | 0.230 |
| ≥2 ultrasound lesions | 52/102 (50.98%) | 65/131 (49.62%) | 0.027 |
| Ultrasound lesion size >5 cm | 35/102 (34.31%) | 60/131 (45.80%) | 0.236 |
| Internal blood flow signal | 70/102 (68.63%) | 103/131 (78.63%) | 0.228 |

*Absolute SMD values greater than 0.10 suggest potentially meaningful imbalance in this non-randomized comparison.*
